# Association between the rate of fluoroquinolones-resistant gram-negative bacteria and antibiotic consumption from China based on 145 tertiary hospitals data in 2014

**DOI:** 10.1186/s12879-020-04981-0

**Published:** 2020-04-07

**Authors:** Ping Yang, Yunbo Chen, Saiping Jiang, Ping Shen, Xiaoyang Lu, Yonghong Xiao

**Affiliations:** 1 Department of Pharmacy, The First Affiliated Hospital, College of Medicine, Zhejiang University, Hangzhou, China; 2 State Key Laboratory for Diagnosis and Treatment of Infectious Diseases, The First Affiliated Hospital, College of Medicine, Zhejiang University, 79 Qingchun Road, Hangzhou, China

**Keywords:** Fluoroquinolones-resistant, *Escherichia coli*, *Klebsiella pneumoniae*, *Pseudomonas aeruginosa*, *Acinetobacter baumannii*, Antibiotic consumption

## Abstract

**Background:**

The purpose of the study is to discuss the correlation between the resistance rate of gram negative bacteria to fluoroquinolones (FQ) and antibiotic consumption intensity of 145 China tertiary hospitals in 2014.

**Methods:**

This retrospective study adopted national monitoring data from 2014. Each participating hospital required to report annual consumption of each antibiotic, and the resistance rate of gram negative bacteria to FQ. Then the correlation between antibiotic usage and fluoroquinolones –resistant (FQR) rate was consequently investigated.

**Results:**

One hundred forty-five hospitals were included in the study, and the median antibiotic consumption intensity was 46.30 (23.93–115.39) defined daily dosages (DDDs) per 100 patient-days. Cephalosporins ranks first in the antibiotics consumption, followed by fluoroquinolones, penicillins, and carbapenems. Fluoroquinolones resistance rate varied from hospital to hospital. The correlation analysis showed significant relationship between the percentage of FQR *Escherichia coli* and the consumption of FQs (r = 0.308, p<0.01) and levofloxacin (r = 0.252, *p*<0.01). For FQR *Klebsiella pneumoniae,* not only FQs (r = 0.291, *p*<0.01) and levofloxacin (r = 0.260, p<0.01) use but also carbapenems (r = 0.242, *p*<0.01) and overall antibiotics (r = 0.247, *p*<0.01) use showed significant correlation. The resistant proportion of FQR *Pseudomonas aeruginosa* was observed to be correlated with the consumption of all antibiotics (r = 0.260, *p*<0.01), FQs (r = 0.319, p<0.01) and levofloxacin (r = 0.377, *p*<0.01). The percentage of levofloxacin-resistant *Acinetobacter baumannii* was significantly correlated with the consumption of all antibiotics (r = 0.282, p<0.01), third-generation cephalosporins excluding combinations with beta-lactamase inhibitors (r = 0.246, p<0.01), FQs (r = 0.254, *p*<0.01) and levofloxacin (r = 0.336, *p*<0.01). However, the correlation of the ciprofloxacin-resistant *A. baumannii* and the antibiotics consumption was not found*.*

**Conclusions:**

A strong correlation was demonstrated between the antibiotic consumption and the rates of FQR gram-negative bacteria. As unreasonable antibiotics usage remains crucial in the proceeding of resistant bacteria selection, our study could greatly promote the avoidance of unnecessary antibiotic usage.

## Background

Fluoroquinolones (FQs) were introduced as broad-spectrum antibiotics. FQs are widely used in infectious diseases due to their excellent oral bioavailability. With the widespread use of FQs, FQs-resistant (FQR) gram-negative bacteria are gradually increasing, limiting the selection for treating infections.

According to China antimicrobial resistance surveillance system in the first half of 2018, the ciprofloxacin-resistant rate was 57.8% for *Escherichia coli*, 35.4% for *Klebsiella* spp., 17.1% for *Pseudomonas aeruginosa* and 75.4% for *Acinetobacter baumannii* respectively. FQ resistance was reported to be an independent risk factor for hospital mortality, which have been associated with greater hospital expenses and poor clinical outcomes [1–5]. Moreover, compared with other phenotypic resistance patterns, bacteremia caused by FQR *E. coli* and *Klebsiella* spp. lead to higher mortality [6]. It is reported that previous colonization of FQR *E. coli* can lead to the spread of extended-spectrum beta-lactamase (ESBL) after the use of quinolone prophylaxis [5, 7]. The high probability of ESBL production by FQR gram-negative bacteria makes anti-infective treatment more difficult. Bloodstream infection caused by FQR gram-negative raised with the increased resistant rate to FQs, and the unadjusted mortality rate was 18% for FQR gram-negative bloodstream infections [1, 8].

It is plausible that the unreasonable antibiotics usage can induce the development of bacterial resistance. In this study, the aim was to investigat the correlation between resistance rate of gram-negative bacteria and antibiotic usage.

## Methods

### Study design

A cross-sectional study involving 145 voluntarily participating hospitals was conducted. Data on antibiotic consumption and the FQR rate of gram-negative bacteria from inpatients at each participating hospital in 2014 were collected. Then the antibiotic consumption and resistant rate was analyzed for possible correlation.

### Data collection

Each participating hospital reported annual data of 2014, including antibiotic consumption and bacterial resistance data. The access to data acquisition can be found in the previous published paper, as well as the source of administrative data of involving hospital [9].

### Measurement of antibiotic consumption and antibiotic resistance

Hospital pharmacists reported each antibiotic consumption to the national antibacterial drug clinical consumption survey network annually. According to Anatomic Therapeutic Chemical (ATC) classification system [10], data on consumption of all antibiotics (J01), beta-lactams (J01C + J01D), beta-lactam-beta-lactamase inhibitor combinations, beta-lactams excluding combinations with beta-lactamase inhibitors (CBLI), penicillins (J01C), penicillins excluding CBLI (J01C-J01CR), cephalosporins (J01DB + J01DC + J01DD + J01DE), cephalosporins excluding CBLI, third-generation cephalosporins (J01DD), third-generation cephalosporins excluding CBLI, fourth-generation cephalosporins (J01DE), carbapenems (J01DH), FQs (J01MA), ciprofloxacin, levofloxacin and moxifloxacin were analyzed. The measurement of antibiotics consumption intensity was the same as our previous published paper [9].

Each participating hospital was required to report information about *E. coli*, *K. pneumoniae, P. aeruginosa* and *A. baumannii.* The information collection of the admitted strains and quality control method of bacteria laboratory in each hospital were the same as our previous report [9]. All hospitals must adhere to the Clinical and Laboratory Standards Institute 2014 guidelines. The FQR rate was defined as the number of FQR isolates divided by the total isolates of the same species tested multiplied by 100. FQs-resistance was defined as a strain resistant to levofloxacin or ciprofloxacin. The FQR rate was considered as the higher resistance rate if the resistance rates were different. For *A. baumannii*, ciprofloxacin and levofloxacin-resistant rate were derived separately because of their great differences. All antibiotic resistance data extraction were performed by the operation of Whonet 5.6 software.

### Statistical analysis

With pearson’s correlation performance, the correlation between annual antibiotic consumption and FQR rate of gram-negative bacteria from 145 hospitals of China was conducted. Through the conduction of Microsoft Excel 2013 and STATA 16 (StataCorp LLC, Texas, USA), the correlation analysis was realized between the FQR rate of gram-negative bacteria and the antibiotic consumption intensity. Statistical significance was defined as *p* < 0.05.

## Results

### Participating hospitals

Altogether 145 hospitals were included in this study, 141 of the participating hospitals were tertiary hospitals. There are 29 hospitals in North China, 29 hospitals in East China, 22 hospitals in Central China, 25 hospitals in Southern China, 12 hospitals in the southwest of China, 15 hospitals in the northwest of China, and 13 hospitals in northeast of China. These hospitals had a median of 2356 beds (range, 720–8475 beds). The median number of inpatients per year reached 88.3 thousands (range, 10–410 thousands).

### Antibiotics consumption

The median of the overall antibiotics consumption intensity in the included hospital was 46.30 DDDs/100 patient-days (23.93–115.39 DDDs/100 patient-days). Among them, cephalosporins were the most- prescribed antibiotics, followed by fluoroquinolones, penicillins and carbapenems. The antibiotics consumption intensity of the main antibiotic classes was depicted in Table 1.

**Table 1:** Antibiotics consumption intensity for the main classes of antibiotics in 145 hospitals

Class (ATC category)	Antibiotics consumption intensity, median value (range, DDDs per 100 patient-days)
All antibiotics(J01)	46.30 (23.93–115.39)
Beta-lactams(J01C + J01D)	33.03 (19.35–67.05)
Beta-lactam-beta-lactamase inhibitor combinations	7.34 (0.73–34.33)
Beta-lactams excluding CBLI	25.95 (13.48–58.82)
Penicillins(J01C)	5.69 (0.99–23.21)
Penicillins excluding CBLI	2.40 (0.02–21.89)
Cephalosporins (J01DB + J01DC + J01DD + J01DE)	24.70 (10.81–52.52)
Cephalosporins excluding CBLI	19.82 (9.20–51.38)
3-GC(J01DD)	10.99 (2.57–38.98)
3GC excluding CBLI	5.99 (1.05–20.99)
4-GC(J01DE)	0.30 (0–5.98)
Carbapenems(J01DH)	1.95 (0.17–10.06)
Fluoroquinolones(J01MA)	5.70 (1.58–19.25)
Ciprofloxacin (J01MA02)	0.09 (0–2.80)
Levofloxacin (J01MA12)	3.73 (0.01–13.56)
Moxifloxacin (J01MA14)	1.41 (0–6.74)

*ATC* Anatomic Therapeutic Chemical; *DDDs* defined daily dosages; *CBLI* combinations with beta-lactamase inhibitors; *3-GC* the third generation cephalosporins; *4-GC* the fourth generation cephalosporins.

### Correlation between antibiotics consumption intensity and FQR *E. coli*

**Fig. 1:**
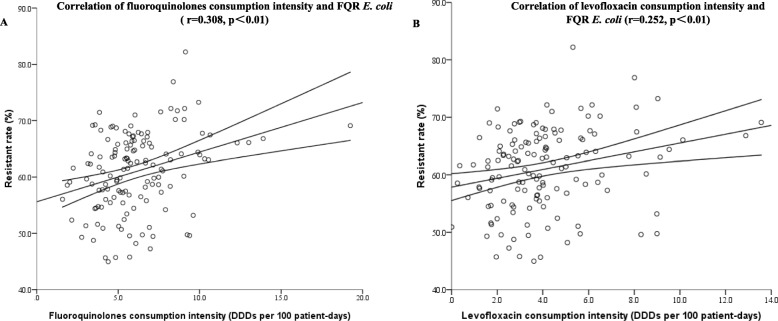
Correlation of FQR *E. coli* and consumption intensity of (**a**) fluoroquinolones; (**b**) levofloxacin

One hundred thirty-eight hospitals were admitted to perform the correlation between antibiotics consumption intensity and rate of FQR *E. coli*. One hundred fifty-eight thousand eight hundred sixty-six strains of *E. coli* were isolated, and 94,247 strains of them were FQR. The median resistance rate of *E. coli* to FQ was 61.67% (44.96–82.2%). As shown in Table 2 and Fig. 1, the resistance rate of *E. coli* to FQ was positively correlated with the consumption of FQs (r = 0.308, *p*<0.01) and levofloxacin (r = 0.252, *p*<0.01).

**Table 2:** Correlations between main classes of antibiotics consumption intensity and the rate of fluoroquinolone-resistant gram-negative bacteria

Classes (ATC category)	Correlation									
	fluoroquinolone-resistant *E. coli* *n* = 138		fluoroquinolone-resistant *K. pneumoniae* n = 139		fluoroquinolone-resistant *P. aeruginosa* *n* = 139		Levofloxacin-resistant *A. baumannii* *n* = 131		Ciprofloxacin-resistant *A. baumannii* *n* = 133	
	*r*^*a*^	p^*^	*r*	*p*	*r*	*p*	*r*	*p*	*r*	*p*
All antibiotics(J01)	0.174	0.062	0.247*	0.003	0.260*	0.002	0.282*	0.001	0.194	0.055
Beta-lactams(J01C + J01D)	−0.035	0.682	0.116	0.173	0.109	0.202	0.165	0.060	0.165	0.058
Beta-lactam-beta-lactamase inhibitor combinations	−0.176	0.093	0.135	0.113	−0.046	0.589	−0.039	0.656	0.037	0.671
Beta-lactams excluding CBLI	0.118	0.167	0.088	0.303	0.152	0.075	0.200	0.072	0.154	0.077
Penicillins(J01C)	−0.118	0.169	0.040	0.643	0.126	0.140	0.081	0.359	0.036	0.678
Penicillins excluding CBLI	0.004	0.964	0.046	0.590	0.212	0.072	0.215	0.064	0.078	0.371
Cephalosporins(J01DB + J01DC + J01DD + J01DE)	−0.014	0.873	0.068	0.426	0.061	0.478	0.157	0.074	0.185	0.053
Cephalosporins excluding CBLI	0.095	0.269	0.034	0.693	0.091	0.286	0.139	0.112	0.141	0.106
3-GC(J01DD)	−0.055	0.524	0.147	0.085	0.063	0.459	0.194	0.067	0.156	0.073
3GC excluding CBLI	0.093	0.276	0.131	0.125	0.109	0.203	0.246*	0.005	0.133	0.127
4-GC(J01DE)	0.037	0.671	−0.063	0.460	0.044	0.611	−0.182	0.077	−0.028	0.748
Carbapenems(J01DH)	0.129	0.131	0.242*	0.004	−0.065	0.445	−0.003	0.975	0.002	0.983
Fluoroquinolones(J01MA)	0.308**	0.000	0.291*	0.001	0.319*	0.000	0.254*	0.003	0.159	0.068
Ciprofloxacin (J01MA02)	−0.014	0.869	0.075	0.383	0.054	0.532	−0.021	0.811	0.077	0.376
Levofloxacin (J01MA12)	0.252*	0.003	0.260*	0.002	0.377*	0.000	0.336*	0.000	0.157	0.072
Moxifloxacin (J01MA14)	0.131	0.127	0.093	0.278	−0.200	0.088	−0.192	0.081	−0.014	0.875

*ATC* Anatomic Therapeutic Chemical; *CBLI* combinations with beta-lactamase inhibitors; *3-GC* the third generation cephalosporins; *4-GC* the fourth generation cephalosporins.

### Correlation between antibiotics consumption intensity and FQR *K. pneumoniae*

**Fig. 2:**
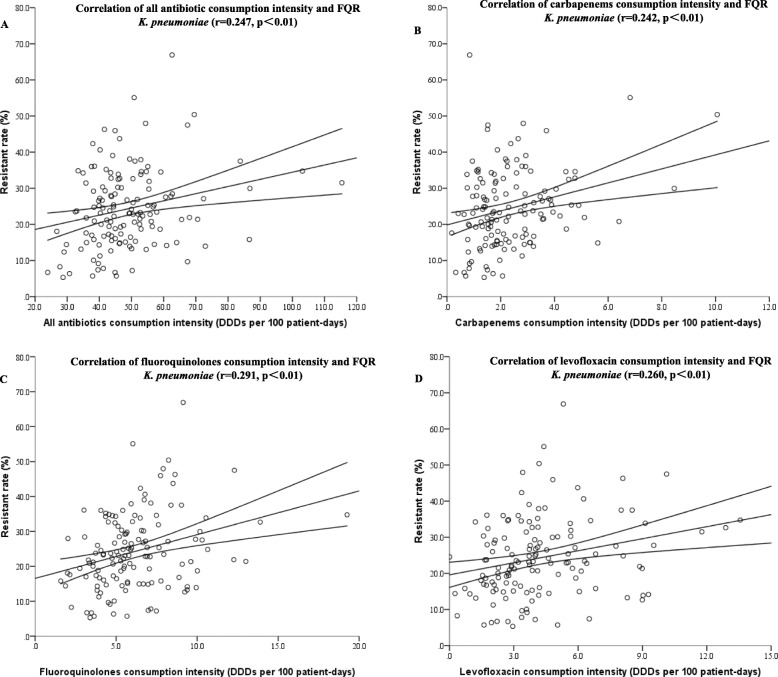
Correlation of FQR *K. pneumoniae* and consumption intensity of (**a**) all antibiotics; (**b**) carbapenems; (**c**) fluoroquinolones; (**d**) levofloxacin

One hundred thirty-nine hospitals were admitted to analyze the correlation between antibiotic consumption intensity and rate of FQR *K. pneumoniae*. One hundred ninety-four thousand nine hundred fifty-seven strains of *K. pneumoniae* were isolated, and 48,287 strains of them were FQR. The median resistance rate of *K. pneumoniae* to FQ was 23.1% (5.3–66.9%). As depicted in Table 2 and Fig. 2, the resistance rate of *K. pneumoniae* to FQ was positively correlated with the consumption of all antibiotics (r = 0.247, p<0.01), carbapenems (r = 0.242, *p*<0.01), FQ (r = 0.291, p<0.01) and levofloxacin (r = 0.260, *p*<0.01).

### Correlation between antibiotics consumption intensity and FQR *P. aeruginosa*

**Fig. 3:**
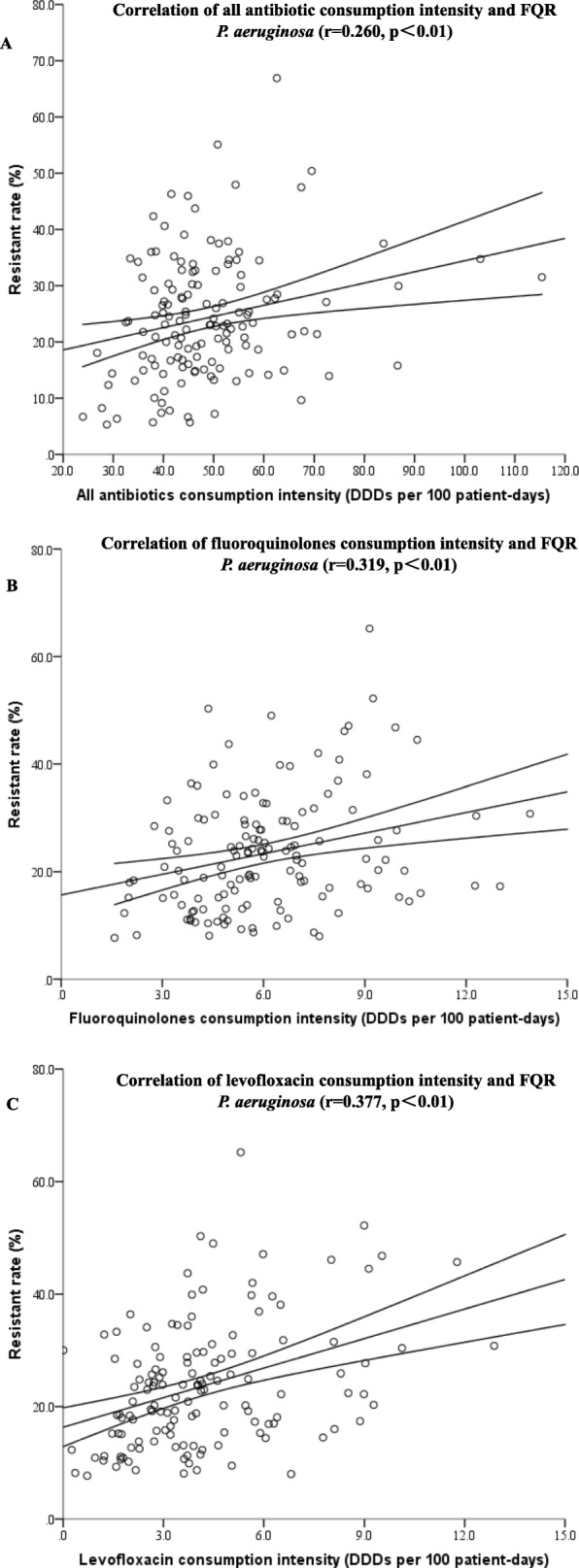
Correlation of FQR *P. aeruginosa* and consumption intensity of (**a**) all antibiotics; (**b**) fluoroquinolones; (**c**) levofloxacin

One hundred thirty-nine hospitals were admitted to determine the correlation between antibiotic consumption intensity and rate of FQR *P. aeruginosa*. One hundred eleven thousand seven hundred eleven strains of *P. aeruginosa* were isolated, and 27,171 strains of them were FQR. The median resistance rate of *P. aeruginosa* to FQ was 22.4% (7.7–65.2%). As demonstrated in Table 2 and Fig. 3, the resistance rate of *P. aeruginosa* to FQ was positively correlated with the consumption of all antibiotics (r = 0.260, *p*<0.01), FQs (r = 0.319, *p*<0.01) and levofloxacin (r = 0.377, *p*<0.01).

### Correlation between antibiotics consumption intensity and FQR *A. baumannii*

**Fig. 4:**
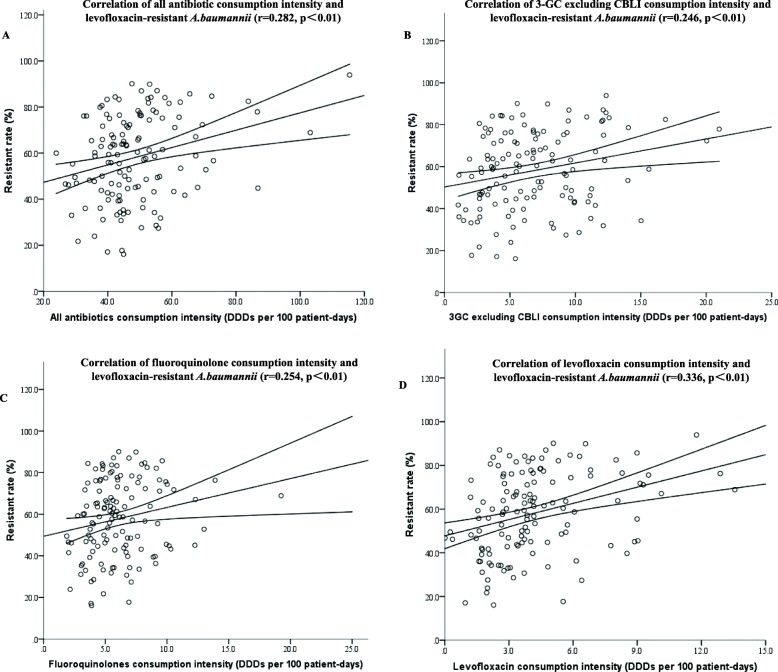
Correlation of FQR *A.baumannii* and consumption intensity of (**a**) all antibiotics; (**b**) combinations of third-generation cephalosporins and beta-lactamase inhibitors; (**c**) fluoroquinolone; (**d**) levofloxacin. 3-GC: the third generation cephalosporins; CBLI: combinations with beta-lactamase inhibitors

One hundred thirty-one hospitals were admitted to conduct the correlation between antibiotic consumption intensity and rate of levofloxacin-resistant *A. baumannii*. Ninety-three thousand one hundred fourteen strains of *A. baumannii* were isolated, and 52,695 strains of them were levofloxacin-resistant. The median resistance rate of *A. baumannii* to levofloxacin was 59.3% (16.1–93.9%). As suggested in Table 2 and Fig. 4, resistance rate of *A. baumannii* to FQ was positively correlated with the consumption of all antibiotics (r = 0.282, *p*<0.01), third-generation cephalosporins excluding CBLI (r = 0.246, *p*<0.01), FQs (r = 0.254, *p*<0.01) and levofloxacin (r = 0.336, *p*<0.01)..

One hundred thirty-three hospitals were admitted to analyze the correlation between antibiotic consumption intensity and rate of ciprofloxacin-resistant *A. baumannii*. One hundred one thousand three hundred seventy-four strains of *A. baumannii* were isolated, and 80,032 strains of them were ciprofloxacin-resistant. The median resistance rate of *A. baumannii* to ciprofloxacin was 77.6% (28.7–95%). However, as shown in Table 2, the percentage of ciprofloxacin-resistant *A. baumannii* was not significantly correlated with the antibiotics consumption..

## Discussion

This study reflected the current status of antibiotic usage and gram-negative bacilli of fluoroquinolones-resistant patterns at the hospital level in China. During the study period, cephalosporins were the most- prescribed antibiotics, followed byfluoroquinolone, penicillins and carbapenems. Our data demonstrated that the percentage of FQR *E. coli* was significantly correlated with the consumption of FQs and levofloxacin while the rate of FQR *K. pneumoniae* was significantly correlated with the consumption of all antibiotics, carbapenems, FQs and levofloxacin. When it comes to FQR *P. aeruginosa,* the resistant rate was significantly correlated with the consumption of all antibiotics, FQs and levofloxacin. Furthermore, the percentage of levofloxacin-resistant *A. baumannii* was significantly correlated with the consumption of all antibiotics, third-generation cephalosporins excluding combinations with beta-lactamase inhibitors, FQs and levofloxacin. However, the correlation of ciprofloxacin-resistant *A. baumannii* and the antibiotics consumption was not found*.*

Consistant with previous studies, our results found that the FQR *E. coli* was significantly associated with FQs consumption [10–19]. The most important resistance mechanism of *E. coli* to quinolones is mutation of target gene DNA gyrase and topoisomerase IV. Further, up expression of active efflux pump, changes in membrane permeability of bacteria and plasmid-mediated quinolone resistance are the main mechanism of FQR *E. coli* production [20]. However, the emergence of FQR *E. coli* occurs as a multistep process, with increasing numbers of target gene mutations leading to progressively higher minimum inhibitory concentration s[21]. Not surprisingly, the most important risk factor for FQR *E. coli* appears to be previous FQs use [22–24]. Restriction of quinolone use can offer opportunities to reduce the prevalence of FQR *E. coli* [25–27].

However, data from 42 Spanish hospitals collected by the European Antimicrobial Surveillance Network indicated that amoxicillin/clavulanic acid use was the main driving force for the progression of FQR *E. coli*, possibly due to its high consumption in Spain [13]. Thirty-six acute care hospitals from France indicated the level of first, second and third-generation cephalosporins, as well as tetracycline’s usage influenced the incidence of FQR *E. coli* [15]. Compared with the third-generation cephalosporins, the resistance rate of *E.coli* to fluoroquinolones is higher, so the third-generation cephalosporins will be extensively used when the infection of *E.coli* is suspected. The author believe that the reason for the correlation between FQR *E. coli* and tegacycline use is that FQR *E. coli* is plausibly associated with resistance to tetracyclines.

There is mounting evidence demonstrating that the prevalence of ciprofloxacin-resistant *K. pneumoniae* was associated with use of ciprofloxacin and FQs [18, 27, 28]. A study from database of the Korean Health Insurance Review and Assessment Service suggested that the consumption of all third-generation cephalosporins was significantly correlated with resistance rates of *K. pneumoniae* to levofloxacin with a quarter lag [29]. The resistance mechanism of *K. pneumoniae* to FQs is the change of target sites, the change of outer membrane protein permeability, the effect of efflux pump and the transfer of resistant plasmids among bacteria. Our study results suggested that the percentage of FQR *K. pneumoniae* was significantly correlated with the consumption of all antibiotics, carbapenems, FQs and levofloxacin. The use of FQs is a risk factor for the incidence of FQR *K. pneumoniae* [30]. Therefore, it is no doubt that the overuse of FQs increases FQR *K. pneumoniae*.

The drug resistance of *K. pneumoniae* is mostly mediated by plasmids. Plasmid DNA can carry multiple drug-resistant genes such as qnr, ESBLs, Amp C enzyme and metalloenzyme-coded genes at the same time. As a mobile genetic primitive, plasmids can transmit carbapenemase-producing resistance genes, resulting in the outbreaks of multidrug-resistant *K. pneumoniae* [31]. Therefore, it is credible that the production of multidrug-resistant *K. pneumoniae* was correlated with the use of carbapenems and all antibiotics.

Resistance to fluoroquinolones developed in *P. aeruginosa* by various mechanisms: mutations in the genes encoding bacterial DNA topoisomerase II and topoisomerase IV is a major cause of resistance to fluoroquinolones in *P. aeruginosa* isolates. Meanwhile, overexpression of active efflux systems can reduce the permeability of the membrane. Furthermore,it is reported that plasmid carrying ESBL gene can also simultaneously carry quinolones resistance gene, which may be the reason of high level of resistance to quinolones in ESBL-producing strains [32].

Consistent with our study, the positive correlation between FQs consumption and the rates of FQR *P. aeruginosa* was described [18, 27, 33–35]. Moreover, the increased consumption of levofloxacin was explored to have correlation with raised resistance rate of *P. aeruginosa* to FQ but not ciprofloxacin [36–40]. Compared with ciprofloxacin, levofloxacin has weaker anti- *P. aeruginosa* activity, and it is easier to screen out the colonization or infection of FQR *P. aeruginosa.* However, various studies indicated that the consumption of ceftazidime, anti-pseudomonal cephalosporin and ciprofloxacin was positively correlated with the incidence rates of ciprofloxacin-resistant *P. aeruginosa* [35, 38, 41]. As we known, the use of certain antibiotics could be both a cause and a consequence of the resistance emergence. So it seems reasonable to explain high consumption of ceftazidime, anti-pseudomonal cephalosporin and ciprofloxacin in hospitals is likely to be a result of the high prevalence of FQR organisms and the alternative drugs usage to treat infection.

*A. baumannii* has become a difficult bacteria in clinical treatment because of its complex drug resistance mechanism and high drug resistance rate. The use of wide spectrum antibiotics will further screen out multidrug-resistant bacteria. Our study showed that the resistance rate of *A. baumannii* to ciprofloxacin was higher than that of levofloxacin, probably because ciprofloxacin is easier to detect the overexpression of pumps [42]. Our study illustrated the selection pressure of FQs use in the development of FQR *A. baumannii*, which is in accordance with a nationwide multicenter study from Kore a[18]. The drug resistance mechanism of *A. baumannii* to quinolones modified the bacterial DNA helix enzyme through the mutation of quinolone resistance gene cluster, thus reducing the affinity between the drug and the enzyme-DNA complex and leading to drug resistance. Also, some efflux systems affect the drug sensitivity. Up to now, three types of plasmid-mediated quinolone resistance genes have been identified, namely aminoglycoside acetyltransferase AAC (6′) - Ib-cr, specific efflux systems Qep A and Oqx AB and Qnr family [43]. Furthermore, simultaneous mutations in *gyrA* and *parC* genes have great influence on high-level fluoroquinolone resistance in *A. baumannii* [44].

Amp C enzyme is a cephalosporin enzyme encoded by chromosomes inherent in all *A. baumannii*. Adding the promoter insertion sequence ISAba1 beside the Amp C gene increases the production of beta-lactamase, which leads to resistance to cephalosporins. This also explains the correlation between drug resistance and consumption of 3GC excluding CBLI [45]. A domestic research report that the consumption of carbapenem has a significant positive relationship with *A. baumannii* resistance to levofloxacin [46]. A retrospective study manifested the consumption of cefmetazole and total cephamycin positively correlated with the resistance rates of *A. baumannii* to levofloxacin. The author believed that these consequences may be partly due to production of AmpC enzymes [47].

With the extensive use of FQs, their adverse reactions and the harm caused by irrational use have become increasingly prominent in recent years. Therefore, drug regulatory agencies at home and abroad frequently issued drug safety warnings, demanding the discontinuation or restriction of the FQs use. In our survey, the resistant rates of all gram-negative bacteria were significantly correlated with the FQs consumption, which is consistant with previous study. It is more likely that the resistance mechanism of gram-negative bacteria to FQs leads to a correlation between resistance and FQs consumption. Therefore, in view of the adverse reactions of FQs and the increasing drug resistance, controlling FQs consumption should be taken seriously.

Antibiotic use is explored to have correlation with the development of antibiotic resistance but the relationship is a multifaceted issue such as cross-transmission, interhospital transfer of resistance, a community contribution to resistance and so on. In our study, the correlation analysis between FQs use and FQ-resistant rate was based on hospital-level perspective, not that of the patient. Therefore, the results may be subject to ecological bias, suggesting that this study dose not reflect patient-level relationships. Additionally, this study did not include the use of outpatient antibiotics, which would underestimate the magnitude of association between FQs exposure and resistant rate to FQ. At the same time, if we can know the phenotype of the resistant isolates, we can definitely know whether any outbreak of cloned strains has affected our research results. If clonal dissemination of resistant strains contributed in part of the resistant organisms present, then a weaker association between antibiotic exposure and resistance might be found. While we acknowledge that our research did not take chronology into account, which is an inevitable defect of the design. This may also be the reason for the not very high correlation coefficient. Even so, it must be stated that the scale of the study made it the most comprehensive investigation reported domestically.

## Conclusion

To summarize, our study quantified the associations between antibiotic consumption and the incidence of FQR gram-negative bacteria. As prudent and appropriate use of antibiotics plays a vital role in preventing the selection of resistant bacteria, our study results could serve as a driving force for implementation of antimicrobial stewardship policies.

## Data Availability

The data used in the current study are available from the corresponding author on reasonable request.

## Abbreviations

*A.baumannii*: *Acinetobacter baumannii*
ATC: Anatomic Therapeutic Chemical
DDDs: Defined daily dosages
*E. coli*: *Escherichia coli*
ESBLs: Extended-spectrum beta-lactamase
FQs: Fluoroquinolones
FQR: fluoroquinolones-resistant
*K. pneumoniae*: *Klebsiella pneumoniae*
*P. aeruginosa*: *Pseudomonas aeruginosa*
